# Supplementary material for: Insulin-like Growth Factor-1 (IGF-1) Related Drugs in Pain Management
Source: Pharmaceuticals (Basel). 2023 May 18;16(5):760. doi: 10.3390/ph16050760 (PMC10224460; doi:10.3390/ph16050760)
Supplement: Supplementary file 1 [file pharmaceuticals-16-00760-s001.zip › pharmaceuticals-2274544-supplementary.pdf]

Table S1. Summary of evidence scores and implications for recommendation

Grade of Recommendations	Benefit vs Risk and Burdens	Methodological Quality of Supporting Evidence	Implications
1A/strong recommendation, high-quality evidence	Benefits clearly outweigh risk and burdens, or vice versa	RCTs without important limitations or overwhelming evidence from observational studies	Strong recommendation, can apply to most patients in most circumstances without reservation
1B/strong recommendation, moderate quality evidence	Benefits clearly outweigh risk and burdens, or vice versa	RCTs with important limitations (inconsistent results, methodological flaws, indirect, or imprecise) or exceptionally strong evidence from observational studies	Strong recommendation, can apply to most patients in most circumstances without reservation
1C/strong recommendation, low-quality or very low-quality evidence	Benefits clearly outweigh risk and burdens or vice versa	Observational studies or case series	Strong recommendation but may change when higher quality evidence becomes available
2A/weak recommendation, high-quality evidence	Benefits closely balanced with risks burden	RCTs without important limitations or overwhelming evidence from observational studies	Weak recommendation, best action may differ depending or circumstances or patients or societal values
2B/weak recommendation, moderate-quality evidence	Benefits closely balanced with risks and burden	RCTs with important limitations (inconsistent results, methodological flaws, indirect, or imprecise) or exceptionally strong evidence from observational studies	Weak recommendation, best action may differ depending on circumstances or patients or societal values

2C/weak recommendation, low-quality or very low-quality evidence	Uncertainty in the estimates of benefits, risks, and burden; benefits, risk and burden may be closely balanced	Observational studies or case series	Very weak recommendations; other alternatives may be equally reasonable

Abbreviations: RCT; randomized clinical trial.

Table S2. Clinical application of IGF-1 related drugs in pain conditions

Pain Conditions	Level of Evidence	Reference	Type of Study	Sample Size	Results	Dose of the Medication	Side Effects
Patellofemoral tendinopathy	2 B+	Olesen et al[37]	RCT, single center	IGF-1 (n = 19), Placebo (n = 17)	Well tolerated, no benefit	1 mg of IGF-1 one dose weekly, 3 injections total intratendinously.	No serious adverse events were reported.
Painful small fiber predominant neuropathy	2 B+	Windebank et al[38]	RCT, single center	IGF-1 (n = 15), Placebo (n = 19)	Safe, not effective	0.05 mg/kg of IGF-1 twice daily for 6 months, subcutaneously.	A total of 356 adverse events, most common injection site pain. Only one serious event; functional bowel obstruction

Teprotumumab for Thyroid-associated Ophthalmopathy	1 B+	Smith et al[39]	RCT, multicenter	Teprotumumab (n = 43), Placebo (n = 45)	Clinical benefit by reducing proptosis, Clinical Activity Score, and improving QOL	Initial dose of Teprotumumab 10 mg/kg followed by 20 mg /kg for total of 8 infusions intravenously	Hyperglycemia, Serious adverse events 5 of 43 patients. Diarrhea and mental confusion "possibly related"

Abbreviations: RCT; randomized clinical trial, IGF-1; insulin-like growth factor-1, QOL; quality of life

Table S3. Summary of 10 studies regarding IGF-1-related drugs in pain conditions

Reference	Methods	Results	Conclusion
Chen et al[30]	Intrathecal injection of IGF-1R inhibitor and anti-IGF1 neutralizing antibodies to mice with CCI	Both IGF-1R inhibitor and anti-IGF-1 neutralizing antibodies reduced mechanical allodynia and thermal hyperalgesia in mice with CCI.	IGF-1R antagonism and IGF-1 neutralization alleviated the pain-related behaviors, relieved the mTOR-induced suppression of autophagy, and mitigated neuroinflammation induced by CCI in mice.
Takemura et al[31]	Intra-plantar injection of IGF-1 and IGF-1R inhibitors to rats after plantar incision	IGF-1 increased GRK2 expression in the ipsilateral DRG. IGF-1R inhibitor prevented both the	IGF-1R inhibition leads to failure of spontaneous resolution of hyperalgesia after

		induction of GRK2 and the resolution of hyperalgesia.	tissue injury. Dysregulation of IGF-1-GRK2 signaling might be one of the major pathological conditions leading to the transition from acute to chronic pain after surgery.
Contreras et al[32]	Subcutaneous recombinant IGF-1 injection to mice with chemotherapy-induced neuropathy	IGF-1 prevented the increase in hot-plate latencies and showed significant improvement in nerve fibers in vincristine-treated mice.	Coadministration of IGF-1 with vincristine prevented behavioral and histopathological manifestations of both sensory and motor dysfunction in a dose-dependent fashion.
Morgado et al[33]	Subcutaneous recombinant IGF-1 injection in STZ-diabetic rats	IGF-1 reversed Fos expression to the control levels at the spinal dorsal horn and the VLPAG and prevented the increased levels of serotonin at the spinal cord and the RVM in STZ diabetic rats.	IGF-1 prevented the behavioral signs of PDN and reversed the neuronal hyperactivity and neurochemical changes at the spinal cord and at the brainstem.

Bitar et al[34]	Intrathecal IGF-1 injection in STZ-diabetic rats	IGF-1 elevated the nociceptive threshold over saline-treated animals by about 35%. mRNA transcripts for IGF-1 and its receptor in the spinal cord were reduced in STZ-diabetic rats, which showed a reduced nociceptive threshold.	The attenuation in the ability of IGF-1 to elevate the nociceptive threshold may be a consequence of reduced gene expression of the IGF-1 receptor within the spinal cord.
Li et al[35]	Intraperitoneal IGF-1 and IGF-1R inhibitor injection in rats with MRMT1 bone cancer pain	IGF-1 increased the expression and function of TRPV1 and significantly increased capsaicin-induced currents in DRG neurons. IGF-1R inhibitor significantly alleviated pain behaviors.	An enhanced TRPV1 function via IGF-1 upregulation in metastasized bone cancer pain and IGF-1 upregulation on TRPV1 through IGF-1R contributes to cancer pain.
Forster et al[36]	Macrophage IGF-1 receptor inhibitor (Linsitinib) was injected through oral gavage in mice with endometriosis.	IGF-1 receptor inhibitor reverses the pain behavior observed in mice with endometriosis	Therapies that modify macrophage phenotype may be attractive therapeutic options for the treatment of women

			with endometriosis-associated pain
Olesen et al[37]	Intratendinous IGF-1 injection in patients with patellar tendinopathy	No significant difference in VAS score, VISA-P score, or biochemical effect compared to the control group	Intratendinous IGF-1 injections with HSR training were safe and well tolerated but did not show any additive improvement in tendon healing compared with resistance training alone
Windebank et al[38]	Subcutaneous IGF-1 injection in patients with painful distal and symmetric neuropathy	No significant difference in the analog pain scale. CASE vibratory threshold favored the placebo group while WBPI walking ability favored the IGF-1 treatment group	IGF-1 can be safely given to patients but may not be beneficial in treating painful small fiber predominant neuropathy

Smith et al[39]	Intravenous IGF-1R inhibitory monoclonal antibody (teprotumumab) injection in patients with thyroid-associated ophthalmopathy	Marked improvement in the primary outcome measure, time to the first response and onset of the response, Clinical Activity Score, proptosis, and GO-QOL visual functioning score in the teprotumumab group	Teprotumumab can safely provide clinical benefit in patients with active, moderate to severe associated ophthalmopathy

Abbreviations: IGF-1; insulin-like growth factor-1, IGF-1R; insulin-like growth factor-1 receptor, CCI; chronic constriction injury, mTOR; mechanistic target of rapamycin, GRK2; G-protein coupled receptor kinase-2, DRG; dorsal root ganglion, STZ; streptozocin, VLPAG; ventrolateral periaqueductal gray, RVM; rostroventromedial medulla, PDN; peripheral diabetic neuropathy, MRMT-1; rat mammary gland carcinoma cells-1 , TRPV-1; transient receptor potential vanilloid subfamily member 1, VAS; visual analog scale, VISA-P; Victorian institute of sport assessment – patella, HSR; heavy slow resistance, CASE; computer assisted sensory examination, WBPI; Wisconsin brief pain inventory, GO-QOL; Graves ophthalmopathy specific quality of life questionnaire

Table S4. Summary of IGF-1R Inhibitors in Pain Conditions

Pain Conditions	Reference	Medication	Result	Conclusion
Chronic Constriction Injury (animal)	Chen et al[30]	IGF1R inhibitor (nvp-aew541)<br>Anti-IGF1 neutralizing antibody	Both IGF-1R inhibitor and anti-IGF-1 neutralizing antibodies reduced mechanical allodynia and thermal hyperalgesia	IGF-1R antagonism and IGF-1 neutralization alleviated the pain-related behaviors, relieved the mTOR-induced suppression of autophagy, and mitigated

			in mice with CCI.	neuroinflammation induced by CCI in mice.
Metastatic bone cancer pain (animal)	Li et al[35]	IGF1R inhibitor (PPP)	IGF-1 increased the expression and function of TRPV1 and significantly increased capsaicin-induced currents in DRG neurons. IGF-1R inhibitor significantly alleviated pain behaviors.	An enhanced TRPV1 function via IGF-1 upregulation in metastasized bone cancer pain and IGF-1 upregulation on TRPV1 through IGF-1R contributes to cancer pain.
Endometriosis (animal)	Forster et al[36]	IGF1R inhibitor (Linsitinib)	IGF-1 receptor inhibitor reverses the pain behavior observed in mice with endometriosis.	Therapies that modify macrophage phenotype may be attractive therapeutic options for the treatment of women with endometriosis-associated pain.
Thyroid-associated	Smith et al[39]	IGF1R inhibitor (teprotumumab)	Marked improvement	Teprotumumab can safely provide

ophthalmopathy (human)			in the primary outcome measure, time to the first response and onset of the response, Clinical Activity Score, proptosis, and GO-QOL visual functioning score in the teprotumumab group	clinical benefit in patients with active, moderate to severe associated ophthalmopathy.

Abbreviations: IGF-1; insulin-like growth factor-1, IGF-1R; insulin-like growth factor-1 receptor, CCI; chronic constriction injury, mTOR; mechanistic target of rapamycin, PPP; picropodophyllotoxin, TRPV-1; transient receptor potential vanilloid subfamily member 1, DRG; dorsal root ganglion, GO-QOL; Graves ophthalmopathy specific quality of life questionnaire
